# The Influence of Sunscreen Use on Skin Pigmentation Disorders: Melasma, Post-Inflammatory Hyperpigmentation, and Vitiligo

**DOI:** 10.3390/cimb48090921

**Published:** 2026-09-09

**Authors:** Bruna Azevedo, Margarida Lorigo, Elisa Cairrao

**Affiliations:** 1 FCS-UBI, Department of Medical Sciences, Faculty of Health Sciences, University of Beira Interior, Av. Infante D. Henrique, 6200-506 Covilhã, Portugal; a46481@fcsaude.ubi.pt; 2 RISE-Health, Faculty of Health Sciences, University of Beira Interior, Av. Infante D. Henrique, 6200-506 Covilhã, Portugal

**Keywords:** skin pigmentation disorders, sunscreen, sun-induced pigmentation, post-inflammatory hyperpigmentation, melasma, vitiligo

## Abstract

Skin pigmentation is influenced by multiple factors, with sun exposure being one of the most important. Therefore, sun protection plays a central role in the prevention and treatment of pigmentation disorders. This work analysed the recent literature (2015–2025) on the influence of sun protection in melasma, post-inflammatory hyperpigmentation, and vitiligo, highlighting therapeutic advances and the impact of sunscreen use in both the prevention and treatment of these conditions. A search was conducted in the PubMed and SCOPUS databases using MeSH terms, selecting original research studies in English and employing various methodologies. Conventional sun protection (UVB/UVA) may not be sufficient on its own to prevent skin pigmentation disorders, given the evidence supporting a role for UVA1 radiation and blue light (380–455 nm) in inducing hyperpigmentation, especially in individuals with phototypes IV-VI. Innovative, broad-spectrum sunscreens, e.g., iron oxide and methoxypropylaminocyclohexenylidene ethoxyethylcyanoacetate, have shown potential for enhancing protection against hyperpigmentation in visible light, while some tinted formulations may also provide cosmetic benefits by camouflaging existing dark spots. The discrepancy between the application doses used compromises observed efficacy, reinforcing the need for public education on photoprotection and for research conducted under real-use conditions. Personalised strategies that combine broad-spectrum and visible-light protection with antioxidant and anti-inflammatory agents may optimise the prevention and management of these disorders.

## 1. Introduction

Skin pigmentation, which means the amount of melanin produced, determines skin colour. There are two main types of melanin—eumelanin and pheomelanin—both produced by melanocytes present in the epidermis [1]. Pheomelanin has a lighter tone, between yellow and red, and does not absorb solar rays as effectively as eumelanin. For this reason, people with higher levels of pheomelanin tend to have lighter skin tones and are more prone to photodamage and sunburn. Importantly, pheomelanin acts as a pro-oxidant; upon UV exposure, it generates reactive oxygen species (ROS) and contributes to oxidative DNA damage, thereby actively increasing the risk of melanoma [2]. Eumelanin, in turn, has a dark brown hue and is highly effective at absorbing solar radiation. Higher levels of eumelanin are associated with darker skin tones and greater protection against solar radiation. Studies also show that high levels of eumelanin are associated with a lower risk of developing melanoma, compared to pheomelanin [2].

The rate of melanin synthesis varies widely, even among members of the same family and racial groups, due to genetic factors, exposure to solar radiation, and hormonal factors such as adrenocorticotropic hormone, lipotropin, and melanocyte-stimulating hormone [2]. Although skin sensitivity to solar radiation does not depend exclusively on the type of melanin present, this is a predominant factor. For this reason, several scales classify skin by its response to sun exposure. One of the most widely used classifications worldwide is the Fitzpatrick phototypes, which comprise 6 types (I-VI) according to genetic predisposition (eye, hair, and photoprotected skin colour), reaction to sun exposure, and tanning habits. This scale has some limitations, as it is subjective and does not consistently correlate with the minimal erythemal dose (the minimum amount of UV radiation required to cause visible erythema on the skin after 24 h of exposure). However, it remains the most used in the current literature [3].

Skin pigmentation disorders can be classified as hyperpigmentation, characterised by increased skin pigmentation, and hypopigmentation, characterised by reduced skin pigmentation. These changes in pigmentation are mainly due to three factors: genetics, exposure to solar radiation and medication. Among the disorders most observed in clinical practice are hyperpigmentation conditions, such as melasma and post-inflammatory hyperpigmentation, and hypopigmentation conditions, such as vitiligo [2]. Among the conditions causing hyperpigmentation, melasma is particularly noteworthy, manifesting as light-to-dark brown patches on sun-exposed areas [4]. Post-inflammatory hyperpigmentation may arise following inflammatory skin processes, dermatological procedures, or other external stimuli, due to increased melanin synthesis or abnormal melanin distribution [5]. In vitiligo, the most common cause of depigmentation, white patches result from the selective destruction of functional melanocytes [6].

Given the recognised influence of solar radiation on the development of skin pigmentation disorders, photoprotection is essential for prevention. However, the literature on the relationship between different types of sunscreens and photoprotection remains unclear. In this sense, this literature review aims to explore the latest advances regarding the relationship between sunscreen use and skin pigmentation disorders. More specifically, sun-induced pigmentation, melasma, post-inflammatory hyperpigmentation, and vitiligo will be the focus. The goal is to identify the impact of sunscreen use in preventing these conditions and in treating pre-existing lesions. It also aims to analyse the latest advancements in their formulations, including new filters and technologies, and reflect on their respective clinical benefits and limitations.

## 2. Methods

**Figure 1:**
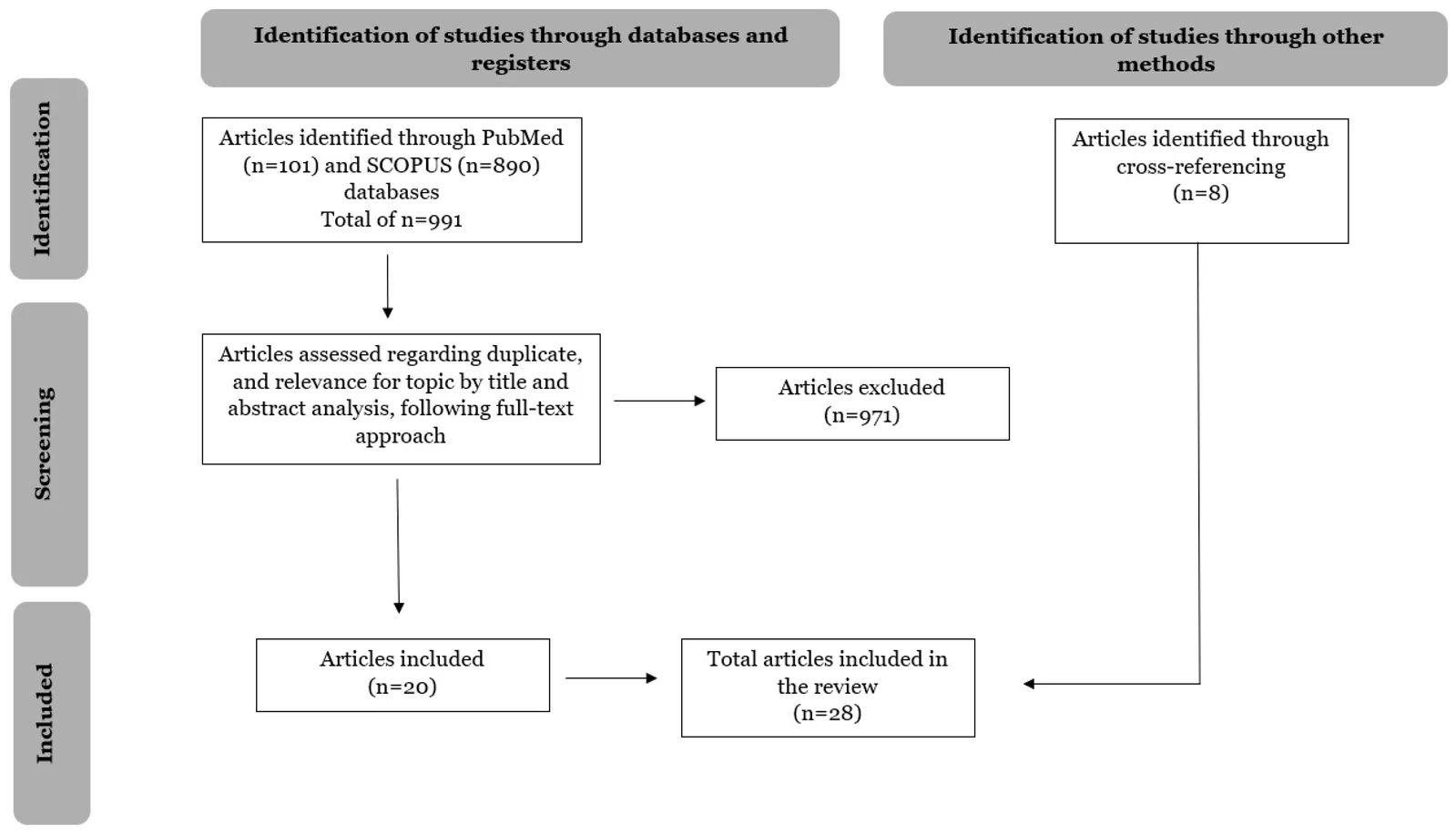
PRISMA Flowchart. Figure created by the authors.

To conduct this Scoping Review, an advanced search was performed on the PubMed platform using the following MeSH Major Topic terms: 1. “radiation” AND “sunscreen”, 2. “hyperpigmentation” AND “sunscreen”, 3. “melasma” AND “sunscreen”, 4. “post-inflammatory hyperpigmentation” AND “sunscreen”; “PIH” AND “sunscreen”, and 5. “vitiligo” AND “sunscreen”. Searches were restricted to articles published between 2015 and 2025 and written in English. Search filters were also applied to exclude review articles, systematic reviews, or meta-analyses. The inclusion criteria were as follows: articles published during the search period (2015–2025); clinical trials, case–control studies, retrospective or prospective studies, longitudinal or cross-sectional studies, laboratory trials; articles written in English; studies relating the use of sun protection to sun-induced pigmentation, melasma, post-inflammatory hyperpigmentation, and vitiligo; and articles relating solar radiation to sun-induced pigmentation, melasma, post-inflammatory hyperpigmentation, and vitiligo. The following exclusion criteria were applied: duplicate articles; review articles, systematic reviews, and meta-analyses; articles published outside the search interval; articles for which the full text was not accessible; articles written in languages other than English; and articles not relevant to the topic. Search 1 resulted in 41 results, 3 of which addressed the relationship between radiation and sunscreen. Search 2 yielded 27 articles, 8 of which were selected that effectively addressed the relationship between sun-induced pigmentation and sunscreen use. As for search 3, it obtained 29 results, 4 of which were selected for the same reason. Search 4 was conducted in two phases: no results were obtained using the MeSH Major Topic term “post-inflammatory hyperpigmentation”; therefore, the term “PIH” was subsequently used, yielding 4 results, 1 of which met the established inclusion criteria. Finally, in search 5, using the MeSH Major Topic terms “vitiligo” AND “sunscreen” yielded no results. Subsequently, a search was performed on the SCOPUS platform using the terms “pigmentation” AND “sunscreen”, and 4 results were related to sunscreen use and its impact on skin pigmentation disorders. This search was also subjected to the aforementioned inclusion and exclusion criteria. Therefore, a total of 101 entries from PubMed and 890 entries from SCOPUS were obtained. The searches were conducted from February to July of 2025. Following the removal of duplicate records, the identified articles were screened by title and abstract. Subsequently, the articles were evaluated through full texts. At this point, it is important to note that access to the full text may have omitted relevant evidence, thereby introducing selection bias; therefore, this limitation was considered when interpreting the results. After the screening and selection of studies was completed, the extracted data included the country, study type, population, sunscreen type, and main results, as reported in the manuscript tables. The entire process was performed by one reviewer and, when necessary, discussed among all authors. Additionally, 8 articles identified through cross-referencing the reference lists of relevant publications were included for their relevance to the topic. These articles were also published between 2015 and 2025 and met the defined eligibility criteria. Finally, 20 original articles identified through PubMed and Scopus database searches, and 8 additional articles identified through cross-referencing, were ultimately included, resulting in a total of 28 original articles analysed. Additional articles published outside this period were included in the review to provide an appropriate mechanistic and historical context and to support the outcomes’ interpretation, but these articles were not included in the 28 articles analysed. This Scoping Review was reported in accordance with the Preferred Reporting Items for Systematic Reviews and Meta-Analyses (PRISMA, Figure 1).

## 3. Solar Radiation

Solar radiation includes ultraviolet (UV), visible, and infrared (IR) radiation. UV radiation can be divided into UVA (320–400 nm), UVB (280–320 nm), and UVC (100–280 nm). Within UVA, there is further subdivision into UVA1 (340–400 nm) and UVA2 (320–340 nm). Because of their different wavelengths, different types of UV radiation exert distinct clinical effects. UVC, with the shortest wavelength, is considered the most harmful to the skin; however, it is completely absorbed by the ozone layer and does not reach the Earth’s surface. UVA, on the other hand, has lower energy than UVB but is approximately 20 times more abundant in the Earth’s atmosphere and is not blocked by glass [7]. The UVA/UVB ratio varies according to season and time of day; UVB intensity peaks around noon, while UVA remains relatively constant throughout the day [8].

Solar radiation causes adverse effects classified as acute or chronic. Acute effects include erythema, pigmentation, suppression of adaptive immunity, stimulation of innate immunity (primarily induced by UVB), and UVA-mediated reduction in blood pressure [8,9]. Chronic effects include photocarcinogenesis and photoaging [10]. UVB is strongly associated with the development of squamous cell carcinoma, and even sub-erythemal doses induce cyclobutane pyrimidine dimers and p53 expression during apoptosis, contributing to photocarcinogenesis [11].

Regarding photoaging, chronic sun exposure results in epidermal thickening, uneven pigmentation, dermal elastosis, collagen degradation, ectatic blood vessels, and increased mutagenesis of keratinocytes and melanocytes [12]. Clinically, this translates to rhytides, telangiectasias, hyperpigmented lesions (lentigines, freckles), volume loss, and cutaneous malignancies. While UVB is predominantly absorbed in the epidermis, UVA penetrates deeper and is the main driver of photoaging. A study of asymmetric facial exposure to UVA reinforced that chronic UVA exposure significantly impacts wrinkle formation [13]. UVA induces these harmful effects through matrix metalloproteinases (MMPs), reactive oxygen species (ROS), and elastases [14]. However, the effects of UVA and UVB often overlap [15].

**Figure 2:**
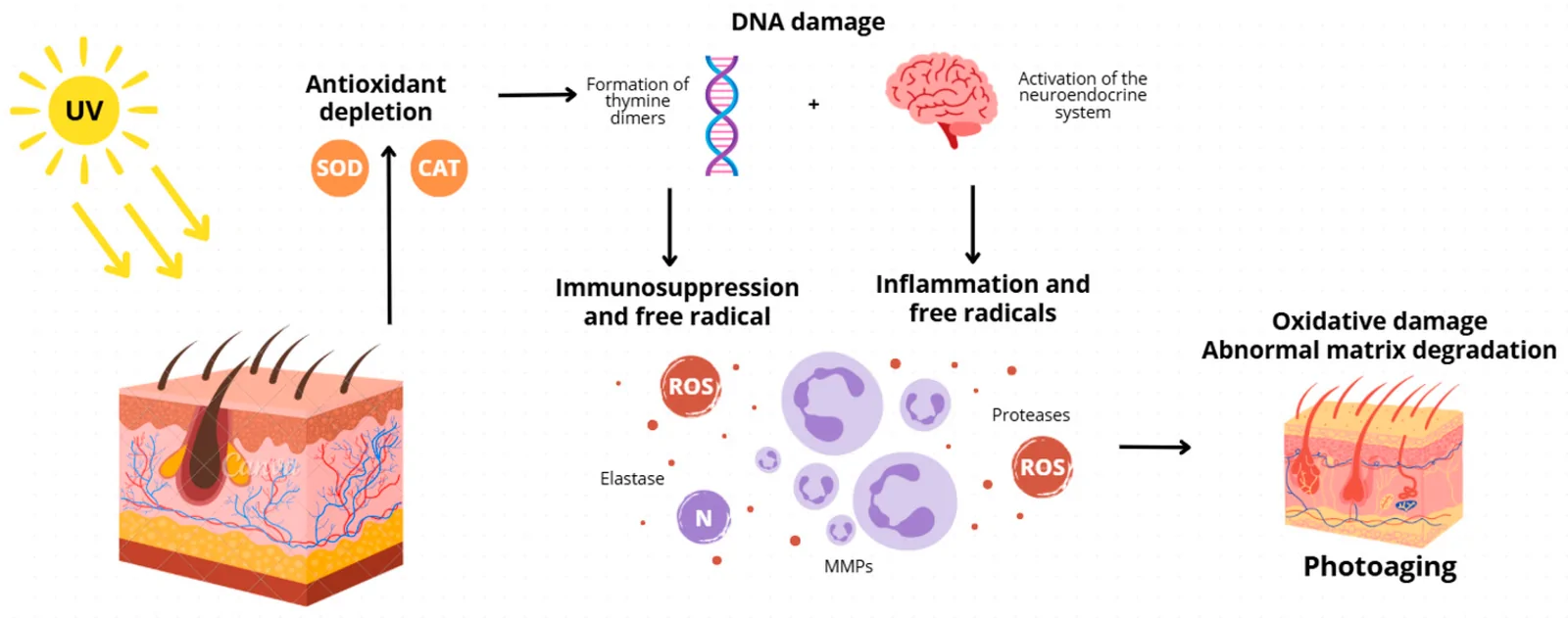
Harmful effects of UV radiation on the skin. Legend: CAT—catalase; DNA—deoxyribonucleic acid; MMPs—matrix metalloproteinases; ROS—reactive oxygen species; SOD—superoxide dismutase; UV—ultraviolet. Figure created by the authors.

In summary, UV radiation induces a cascade of complex biochemical reactions in human skin. Keratinocyte and Langerhans cell deoxyribonucleic acid (DNA) absorbs UV radiation, depleting enzymatic and non-enzymatic antioxidants (e.g., superoxide dismutase and catalase), initiating DNA damage, forming thymidine dimers, and activating the neuroendocrine system, leading to immunosuppression. Additionally, UV promotes the release of pro-inflammatory mediators, increasing capillary permeability and allowing infiltration by neutrophils and phagocytes. This leads to inflammation, ROS generation, and abnormal extracellular matrix degradation. Oxidative stress damages proteins, lipids, and carbohydrates, which accumulate in the dermis and epidermis, contributing to photoaging (Figure 2). The role of infrared radiation (700 nm^−1^ mm) in photoaging and the induction of skin damage is also highlighted. Visible light (400–700 nm), particularly blue light (380–455 nm), has also been implicated in pigmentation; the 380–400 nm portion overlaps with the UVA range. Independently, visible light induces the formation of ROS, pro-inflammatory cytokines, and MMP-1 expression, and enhances the effects of UV radiation [16,17]. Overall, exposure to solar radiation is estimated to account for approximately 80% of the clinical signs of skin ageing [14,18].

## 4. Sun Protection

There are several strategies to prevent UV radiation-induced skin damage. Among these, physical and chemical sunscreens stand out, preventing the cutaneous penetration of UV radiation; anti-inflammatory compounds (such as cyclooxygenase inhibitors and cytokine generation inhibitors), which prevent and reduce inflammation; antioxidants that eliminate ROS produced by UV radiation; retinoids and other synthetic and natural inhibitors, which inhibit neutrophil elastase activity, preventing damage to the extracellular matrix and inhibiting the expression and activity of MMPs [14]. Within this broad spectrum of existing photoprotection methods, the present review will focus specifically on sunscreens.

Sunscreen has clearly demonstrated clinical benefits in preventing the development of actinic keratoses, squamous cell carcinomas, nevi, and melanomas. Furthermore, it also prevents photoimmunosuppression and signs of photoaging [8]. Sunscreens can be divided into organic (chemical) and inorganic (physical). Chemical sunscreens account for about 75% of products on the market and work by absorbing UV radiation and converting it into heat. In contrast, physical sunscreens consist of insoluble metal oxide particles, typically zinc oxide and titanium dioxide, which form an opaque film (with a white, chalky texture) on the skin, reflecting, scattering, and absorbing some of the UV rays [19,20]. One limitation in assessing sunscreen efficacy is that most consumers rely on the sun protection factor (SPF) as the sole indicator of the photoprotection they provide. Even the American Academy of Dermatology and the Canadian Dermatology Association recommend using sunscreen with a minimum SPF of 30. However, SPF only measures the ability of sunscreen to prevent erythema induced by UV radiation, especially UVB, which is about 1000 times more erythemogenic than UVA. Thus, SPF essentially evaluates protection against UVB, without considering the harmful effects of UVA radiation or visible light [19]. In addition, SPF is calculated using a sunscreen quantity of 2.0 mg per cm^2^ of skin, which does not correspond to actual consumer use, typically around 0.39–1.0 mg/cm^2^. This insufficient application considerably reduces the photoprotection offered. Therefore, it is necessary to invest in consumer education, whether it be earlier reapplication of sunscreen, purchasing a higher-SPF sunscreen, or using the indicated amount of sunscreen [21]. Similarly, it is important to note that sunscreens available on the market have minimal effects in reducing ROS induced by visible light, suggesting that these sunscreens, despite protecting against UVA and UVB, do not protect against visible light. Effectively, there is a lack of Food and Drug Administration (FDA)-approved filters that protect against UVA1 (340–400 nm), particularly the long-UVA range above 370 nm, and none are effective against visible light [15]. While standard inorganic UV filters, such as zinc oxide and titanium dioxide, provide broad-spectrum protection against UV radiation, they leave a visible whitish tint on the skin, which can be cosmetically undesirable for consumers [15]. To overcome these cosmetic limitations and target the oxidative stress induced by solar rays and visible light, modern sunscreens incorporate antioxidant additives. Indeed, it has been shown that adding an antioxidant combination to a broad-spectrum sunscreen significantly reduces the production of ROS, inflammatory cytokines, and MMPs in vitro and decreases oxidative stress in vivo [16].

## 5. Sun-Induced Pigmentation

Sun exposure is a common cause of changes in skin pigmentation. In response to solar radiation, the body increases melanin production as a defence mechanism against its harmful effects [2]. UV radiation stimulates keratinocytes—the main cells of the epidermis—to produce free radicals which, together with the radiation itself, activate biological mediators capable of influencing melanocyte activity. This stimulation activates the enzyme tyrosinase, which is responsible for melanin production and converts the amino acid tyrosine into melanin pigments (pheomelanin or eumelanin, depending on intra- and inter-individual factors). Melanin is then transferred to keratinocytes in the form of small granules called melanosomes via melanocyte dendrites, which connect these cells at a ratio of 30 to 40 keratinocytes per melanocyte. As they receive melanin, keratinocytes transport it and migrate to the most superficial layers of the skin over several days. In this way, melanin spreads across the skin’s surface, giving it a darker tone. Through the skin’s natural exfoliation process, the outermost cells are shed, and pigment is gradually lost [22]. Although this mechanism is protective, it can exacerbate pre-existing pigmentation disorders, making it essential for these patients to use sunscreen daily to protect their skin against hyperpigmentation. Ideally, they should opt for broad-spectrum formulations that protect against both UV and visible light. This recommendation is particularly relevant because visible light plays an important role in the induction of hyperpigmentation. Despite this, many sunscreens currently available on the market do not offer adequate protection against this type of radiation [19]. In this regard, sunscreen formulations enriched with iron oxide have been investigated, as this compound provides more comprehensive protection, including against visible light. Furthermore, it offers an additional benefit, as, in addition to preventing the development of sun-induced skin pigmentation, it also conceals existing hyperpigmentation [23]. Choosing the right sunscreen and patient education are therefore essential; one of the greatest challenges in the treatment of pigmentation disorders is the lack of information on sun protection, particularly among individuals with darker phototypes [24].

## 6. Skin Hyperpigmentation Disorders

### 6.1. Melasma

Melasma is a common hyperpigmentation disorder, affecting up to 30% of women of childbearing age. It is characterised by light-to-dark brown hyperpigmented patches with a symmetrical distribution and irregular margins in photoexposed areas. These patches most commonly affect the face, namely the malar area, forehead, and upper lip, with consistent clinical worsening in summer [4]. Melasma can be classified into types (epidermal, dermal, and mixed) based on the level of melanin deposition [25]. Historically, melasma is considered a consequence of female hormone stimulation in a genetically predisposed individual. However, more recent studies identify melasma as a photoaging disorder, a hypothesis supported by histological findings. Alterations of the basement membrane, increased solar elastosis, dermal mast cells, and vascularisation can be found in melasma lesion areas [4,26].

The impact of solar radiation as a predisposing factor for melasma is already known. However, even with sun protection against UVB and UVA, most patients continue to show clinical worsening during the warmer months, suggesting that more comprehensive protection is needed, especially against visible light. Additionally, it has recently been discovered that lower-wavelength visible light (blue–violet) induces greater hyperpigmentation through a specific opsin-3 receptor pathway in melanocytes, activating a calcium signalling cascade that activates the tyrosinase complex. This discovery could have important therapeutic implications in the future [4].

Melasma treatment is often ineffective, with frequent relapses. This occurs because treatment focuses on melanosis and melanocyte changes associated with melasma, while failing to address the photodamaged areas around melanocytes that cause relapses [27]. Currently, the most widely accepted type of treatment is topical bleaching agents, specifically Kligman’s formula (a combination of corticosteroids, retinoid acid, and hydroquinone). Chemical peels and laser therapy can be used; however, results are inconsistent and can lead to post-inflammatory hyperpigmentation. Regardless of the chosen treatment modality, it must be associated with high-index sun protection [4,25]. More recently, it has been shown that oral tranexamic acid taken 2 to 3 times a day for 2 to 3 months decreases the melanin and erythema index in melasma lesions. This compound acts by inhibiting UV radiation-induced melanogenesis and neovascularisation, by blocking plasminogen activation and plasmin activity [28]. It has therefore been proposed to combine traditionally used topical melasma treatments with other products used for photoaging, such as topical tretinoin, tranexamic acid, and energy-based devices, to promote collagen remodelling [4].

### 6.2. Post-Inflammatory Hyperpigmentation

Post-inflammatory hyperpigmentation is a reactive process resulting from increased melanin production or abnormal melanin distribution, secondary to inflammatory skin conditions, dermatological treatments, or external stimuli [5]. Among its most common etiological agents are acne vulgaris, atopic dermatitis, and impetigo. This reaction also occurs very frequently after laser therapy [24,29]. Typically, it is more common in phototypes IV to VI and is also more severe [29]. Lesions are characteristically limited to the sites of previous inflammation and have irregular margins [30]. This condition results from excessive melanin production or irregular melanin distribution following cutaneous inflammation. Inflammatory mediators, such as cytokines and reactive oxygen species, stimulate melanocyte activity. This inflammatory response can be induced by exposure to UV radiation and visible light [24]. When hyperpigmentation is confined to the epidermis, there is an increase in melanin production and transfer to adjacent keratinocytes. These lesions will then have a brown colouration. This epidermal reaction is typically associated with post-inflammatory hyperpigmentation caused by acne (e.g., [29,30]). On the other hand, dermal hyperpigmentation indicates inflammatory damage to basal keratinocytes, with disruption of the basement membrane and the release of large amounts of melanin. This free pigment is then phagocytosed by macrophages (which are then called melanophages) in the uppermost layer of the dermis, producing a bluish-grey skin colouration at the lesion site. This type of post-inflammatory hyperpigmentation is highly associated with lichen planus and cutaneous manifestations of systemic lupus erythematosus [29,30].

The use of photoprotection is an important adjuvant in the treatment and prevention of hyperpigmentation exacerbation [24]. The treatment of post-inflammatory hyperpigmentation is based on managing the underlying inflammatory condition. The first line consists of topical depigmenting agents, such as tyrosinase inhibitors, hydroquinone, and azelaic acid, used in combination with sunscreen. This therapy is usually effective for epidermal hyperpigmentation. However, care must be taken when using these products due to the risk of worsening post-inflammatory hyperpigmentation and skin irritation. Other therapeutic options include chemical peels, laser, and phototherapy [29].

## 7. Skin Hypopigmentation Disorders

### Vitiligo

Vitiligo is a chronic, acquired skin pigmentation disorder, characterised by the formation of well-defined white macules, resulting from the selective destruction of functional melanocytes in both the skin and hair. It frequently affects the face and other exposed areas of the body. It is the most common cause of depigmentation worldwide, affecting about 1% of the population, and has an early onset [6,31]. Vitiligo can be divided into two types—segmental and non-segmental—with different clinical courses, prognoses, and treatments. Non-segmental vitiligo, the most common form, is characterised by bilateral and symmetrical depigmented macules. It often starts with an acrofacial pattern (affecting the face and distal extremities) before progressing to a generalised distribution throughout the entire body, typically affecting extensor surfaces prone to friction and trauma (the Koebner phenomenon). This form of vitiligo has an unpredictable course and sometimes can progress rapidly, within weeks to months. Meanwhile, the segmental type has a unilateral distribution and a quasi-dermatomal distribution that spreads rapidly but stabilises within a few months. Single, isolated depigmented lesions that cannot be categorised at onset are classified as focal vitiligo, and if they do not progress to either of these two forms within 1 to 2 years, they are then considered unclassifiable vitiligo [6]. In histological terms, vitiligo lesions are characterised by an almost complete absence of functional epidermal melanocytes, with active margins showing a CD8+ cytotoxic T-cell lymphocytic infiltrate, although a clinically visible erythematous border is rare and limited to the inflammatory variant [6].

The causes of vitiligo are still not well understood. It has been suggested that this disease has multifactorial causes, both genetic and environmental. Vitiligo is thought to be an immune-mediated disease, as it is intimately associated with other immune-mediated conditions, such as Hashimoto’s thyroiditis, rheumatoid and psoriatic arthritis, adult-onset diabetes mellitus, Addison’s disease, pernicious anaemia, alopecia areata, and systemic lupus erythematosus. There is also a higher incidence of immune-mediated diseases in relatives of people with vitiligo, suggesting that this disorder has a strong genetic component [6]. Conversely, patients with vitiligo have a lower risk of developing skin cancer, especially melanoma. This lower incidence is not fully explained, but it could be attributed to increased dermatological surveillance, greater care and adherence to photoprotective measures, and a lower presence of dysfunctional melanocytes due to the lower absolute number of melanocytes [32,33]. There is also an association between vitiligo and halo nevi (a nevus surrounded by an area of depigmentation). This type of nevus is common in healthy individuals, but they are 8 to 10 times more common in patients with vitiligo. The presence of multiple halo nevi is a cellular marker of autoimmunity against clustered melanocytes and may indicate an increased risk of vitiligo, especially with a family history [31]. Vitiligo involves a chronic cycle of worsening, stabilisation, and repigmentation phases in genetically predisposed individuals. Worsening phases are triggered by environmental factors, such as UV radiation exposure, chemical substances, and microinfections, which induce the production of pathogen- and damage-associated molecular patterns, which interact with the NLRP1 receptor in the cytoplasm of dendritic cells, activating innate immunity and, subsequently, adaptive immunity, culminating in the disappearance of melanocytes [6].

Vitiligo treatment consists of phototherapy, topical treatments with tacrolimus and corticosteroids, and surgical treatment. The choice of the most appropriate treatment depends on several criteria, among which the type of vitiligo (non-segmental vs. segmental), the presence of the Koebner phenomenon (development of vitiligo lesions in healthy skin areas after local trauma), and leukotrichia stand out [31]. Generally, the 1st-line treatment for vitiligo consists of applying topical corticosteroids and calcineurin inhibitors, once daily, discontinuously, to avoid local adverse effects (skin atrophy, telangiectasias, hypertrichosis, acneiform eruptions, and striae) [6]. Meanwhile, 2nd-line treatment is based on phototherapy, in association with systemic corticosteroids. The literature indicates that it is preferable to use narrowband UVB radiation selectively and locally on vitiligo lesions; however, treatment duration and intensity are highly variable. This form of treatment yields much more promising results when initiated in the early stages of disease development [31,34]. Although exposure to natural sunlight has been investigated as a potential adjunctive approach in vitiligo, it should be clearly distinguished from medically supervised phototherapy. Uncontrolled exposure to sunlight, particularly exposure sufficient to induce erythema or sunburn, should not be considered a therapeutic goal because of the risk of acute and cumulative photodamage [32]. Finally, surgical treatment is reserved for patients with segmental or non-segmental vitiligo with stable disease for more than 1 year, unresponsive to medical therapies, and without the Koebner phenomenon [6].

## 8. Results

Sun exposure, particularly UV radiation, has a significant impact on inducing skin pigmentation. In particular, the synergistic relationship between UVA1 rays and visible light intensifies induced pigmentation, reinforcing the need to develop effective photoprotection methods against both [35,36]. In this context, a strong correlation is evident between visible light protection factor and pigmentation protection factor [37]. In fact, protection against visible light plays a crucial role in reducing skin hyperchromias by inhibiting melanin synthesis and promoting cell renewal [38]. Among the solutions under study, sunscreens enriched with iron oxide have shown potential for improving protection against pigmentation induced by visible and UVA1 radiation [39]. Additionally, iron oxide has a dual beneficial effect, acting not only to prevent hyperpigmentation but also to camouflage pre-existing spots [40]. Available evidence suggests that tinted sunscreens may provide greater protection against visible light and UVA1 than colourless formulas [41]. Schalka, S., et al. [42] also applied a spectrophotometric evaluation of 33 sunscreens, introducing visible light protection factor and pigmentation protection factor. Only formulations containing iron oxide provided significant protection (values >3), indicating that protection against visible light is independent of the SPF indicated on the product label. Within the visible light spectrum, blue light warrants special attention due to its significant impact on skin hyperpigmentation [43,44,45]. It has been shown that, within visible light, radiation with shorter wavelengths, such as blue light, can induce prolonged hyperpigmentation, while radiation with longer wavelengths does not affect pigmentation [46]. Furthermore, the UV/visible limit (385–405 nm) induces significant biological damage, including the formation of “dark” cyclobutane pyrimidine dimers in human skin in vivo. Up-regulation of genes associated with inflammation and photoaging (e.g., IL-6 and MMP-1) has been demonstrated, highlighting the importance of sunscreens that provide protection in this range, which is currently a gap [47]. For this reason, several formulations with preventive effects have been developed. Among these, a study demonstrated that incorporating the sunscreen filter phenylene bis-diphenyltriazine in sunscreens prevents immediate skin pigmentation caused by sun exposure [44]. Additionally, *Scenedesmus rubescens* microalgal extract and niacinamide are being investigated for their potential to attenuate this effect, with positive results [43].

In parallel, several studies have evaluated the benefits of adding methoxypropylamino cyclohexenylidene ethoxyethylcyanoacetate (MCE) to sunscreens, a filter that offers coverage across the entire UV spectrum, including UVA1 radiation [37,48,49,50]. Marionnet, C., et al. [48] demonstrated that conventional sunscreen formulations provided effective absorption of UVB and UVA wavelengths up to wavelengths of approximately 360–370 nm, whereas the addition of MCE, a UVA1 filter with a peak absorption at 385 nm, extended the absorption profile toward 400 nm and photoprotection in the UVA1 range. This evidence was reinforced by a randomised prospective study involving 113 women from diverse environmental and genetic backgrounds, which compared the same sunscreen formulation with and without 3% MCE. The results showed that the enriched formulation significantly reduced induced pigmentation [49]. Similar results were obtained by de Dormael, R., et al. [50] and Mercurio, D. G., et al. [37], who tested MCE concentrations of 2% and 1%, respectively. Thus, despite encouraging findings across studies, further investigation is needed to determine whether different concentrations of this sunscreen filter provide differential benefits in preventing hyperpigmentation, while maintaining an adequate safety profile and avoiding side effects. In this regard, it is important to note that the European Union’s Scientific Committee on Consumer Safety defines the use of MCE concentrations up to 3% as safe [48]. The Fitzpatrick phototype is also an important factor to consider when choosing the appropriate photoprotection spectrum. A clinical trial concluded that, after 4 min of simulated sun exposure in the UV and IR spectrum, the formation of free radicals in Fitzpatrick skin types IV and V was 60% of that observed in type II. This result highlights the need for special care in photoprotection for visible light in these phototypes [51]. All epidemiological and in vitro studies presenting a relationship between melasma and sunscreens are summarised in Table 1 and Table 2, respectively.

### 8.1. Melasma

Melasma lesions are associated with sun-induced DNA hypermethylation [53]. Although the enzyme DNA methyltransferase 1 (DNMT1) is overexpressed under these conditions, applying sunscreen containing niacinamide or retinoic acid reduced DNMT1 and DNMT3b methylation and expression levels, which correlated with clinical improvement in the spots. These results highlight the importance of incorporating UV-protection treatments, coupled with DNA repair and genomic stability strategies, into clinical practice [53]. The regular use of SPF 19 and PA+++ (UVA protection grade scale) sunscreen has been associated with improvements in the Melasma Area Severity Index (MASI) and the quality-of-life index for melasma patients [54]. Additionally, iron oxide-enriched tinted sunscreens have shown potential to enhance protection against shorter-wavelength visible light, which has been associated with increased skin pigmentation [46]. Although the use of sunscreen alone has shown positive results, combinations of different active ingredients with sunscreen are being studied to enhance its benefits. A prospective clinical trial with 12 Caucasian women with melasma showed that an SPF 50+ sunscreen, combined with ingredients such as niacinamide, hydroxyphenoxypropionic acid, dipotassium glycyrrhizinate, glycolic acid, and 4-n-butylresorcinol, results in rapid clinical improvement in melasma hyperpigmentation [55]. Chatterjee, M., et al. [56] reported that the combination of an SPF 30 sunscreen with the active ingredients phenylethyl resorcinol, nonapeptide-1, aminoethylphosphinic acid, and antioxidants not only improved the melanin index but also enhanced MASI results and was associated with better maintenance of melasma remission than the isolated use of sunscreen. All epidemiological studies presenting a relationship between melasma and sunscreens are summarised in Table 3.

### 8.2. Post-Inflammatory Hyperpigmentation

Laser therapy is frequently used to treat skin pigmentation disorders, such as solar lentigines and nevi, by removing most melanocytes and melanin from the epidermis. However, laser treatment has post-inflammatory hyperpigmentation as an adverse effect, especially in darker skin phototypes. This phenomenon occurs due to the activation of remaining melanocytes, resulting in hyperpigmentation approximately 1 week after laser exposure [57]. Histopathological studies demonstrate that this hyperpigmentation is specifically due to increased melanogenic activity, and not to the number of melanocytes, and may manifest itself through distinct epidermal or dermal patterns [58]. Therefore, the ideal time to intervene to prevent post-inflammatory hyperpigmentation is within this first week after treatment [57]. Similarly, a clinical trial involving 230 Indian volunteers with skin pigmentation changes (including post-inflammatory hyperpigmentation) demonstrated that regular use of moderate SPF and PA+++ sunscreen not only protects against pigmentary irregularities but also improves skin luminosity in Fitzpatrick phototypes IV and V, attenuating signs of photoaging [59]. In this way, it is believed that the association of sunscreen with anti-inflammatory agents and sebaceous production reducers can enhance the reduction in hyperpigmentation. However, the difference between the results obtained with this combination and those achieved with the isolated use of sunscreen did not show statistical significance, reinforcing the need for more studies to validate this approach [60]. The distinction between these patterns is fundamental for therapeutic success, given that the dermal type presents greater inflammatory infiltration and loss of basement membrane integrity, which may require adjustment of the laser wavelength or the use of specific anti-inflammatory therapies [58]. All epidemiological studies presenting a relationship between post-inflammatory hyperpigmentation and sunscreens are summarised in Table 4.

### 8.3. Vitiligo

Although the exact risk factors associated with the development of vitiligo are not yet fully understood, epidemiological evidence from a prospective cohort study of 51,337 women revealed that individuals with skin marks larger than 3 mm on the upper limbs, a greater tanning capacity, and a history of blistering sunburns face a higher risk of incident disease [61]. Because the loss of functional epidermal melanocytes deprives vitiliginous lesions of their natural UV barrier, the daily use of broad-spectrum sunscreen (SPF ≥ 30) is clinically recommended to prevent acute sunburns, avoid active disease progression through the Koebner phenomenon, and minimise cosmetic contrast with surrounding tanned skin. Regarding treatment, the most used approach involves 2 to 3 weekly sessions of narrow-band UVB phototherapy over a period of 6 to 12 months, with the aim of stimulating skin repigmentation. NB-UVB phototherapy should be distinguished from exposure to natural sunlight, as phototherapy involves controlled ultraviolet radiation exposure with individualised dosing, whereas uncontrolled sun exposure as well as the prolonged use of this therapy may result in erythema, sunburn, and cumulative photodamage. To clarify this issue, a study was conducted with 60,231 patients diagnosed with vitiligo in South Korea, which concluded that prolonged narrowband UVB treatment is not associated with an increased risk of skin cancer and is therefore a safe therapeutic option [62].

## 9. Discussion

Despite decades of research, skin pigmentation disorders are not yet fully understood and involve multiple etiological factors, including genetic factors, sun exposure, local skin trauma, and pharmacological interactions. Still, current evidence points to solar radiation as one of the most important causes for the onset and worsening of these conditions, making sun protection a fundamental step in their prevention and treatment [2].

The results of this review demonstrate that conventional sun protection, focused only on UVB and shorter-wavelength UVA radiation, is not sufficient to prevent or control skin pigmentation disorders. It has been shown that UVA1 radiation and visible light, particularly blue light (380–455 nm), play a significant role in inducing and exacerbating hyperpigmentation [35,36]. This finding is consistent with recent reviews, such as McDonald, K. A., et al. [19] and Passeron, T., et al. [63], which emphasise the need for broad-spectrum formulations that go beyond conventional sunscreen filters. Similarly, Ko, D., et al. [64] reported that UV radiation and visible light exacerbate pre-existing dyschromias, and, for this reason, all individuals with pigmentation disorders should use tinted broad-spectrum sunscreen with an SPF equal to or greater than 30. Furthermore, this review demonstrates that the daily use of broad-spectrum sunscreen, in addition to preventing the appearance of new spots, promotes the lightening of pre-existing spots [64]. Recent studies, directed at Indian populations, have shown that individuals with higher Fitzpatrick phototypes (IV-VI) have a greater predisposition to develop hyperpigmentation induced by visible light and long UVA, compared to the rest of the solar radiation spectrum [59]. More specifically, visible light causes darker and more sustained skin pigmentation in these phototypes, when compared to visible radiation, as supported by Abdel Azim, S., et al. [65]. For this reason, the adoption of adequate photoprotection measures for these wavelengths in these populations becomes especially relevant, as corroborated by [63]. A prospective 12-month study showed that daily use of broad-spectrum SPF 30 sunscreen improves hyperpigmentation and dark spots in 52 individuals with Fitzpatrick phototypes IV to VI, when compared to a group of individuals of the same age and phototype who do not use sun protection [65]. Despite this finding, many sunscreens available on the market focus mainly on UVB and shorter-wavelength UVA radiation, not offering effective protection against visible light and longer-wavelength UVA. Therefore, they are not sufficient to prevent pigmentation changes [15]. Among emerging strategies, tinted sunscreens with iron oxide or coloured pigments have shown potential for improving protection against visible light and may also provide cosmetics benefits by helping to camouflage existing hyperpigmentation [23]. This advantage makes these products especially important for individuals with melasma and post-inflammatory hyperpigmentation. This beneficial effect has already been noted by McDonald, K. A., et al. [19], Passeron, T., et al. [63] and Ko, D., et al. [64]. In addition to this compound, Abdel Azim, S., et al. [65] also highlight the potential beneficial effect of zinc oxide, effective against UVA, and titanium dioxide, effective against UVB, as these compounds absorb UV light and refract and reflect part of the radiation, including visible light [65]. However, it was found that tinted sunscreens cause a visible alteration in skin tone which, combined with their texture, compromises the use of this compound and its long-term adherence [23]. This point reinforces that the clinical efficacy of dermatological products depends not only on their formulation but also on their cosmetic acceptability and suitability for user preferences. Therefore, it is pertinent that, in developing new filters that protect against visible light and the UVA1 spectrum, cosmetic requirements are considered to ensure consumer acceptance.

In the case of melasma, evidence supports the use of sun protection to improve MASI and quality-of-life index scores in patients [54]. The results are even more promising when the sunscreen is tinted and enriched with iron oxide [46]. Also, combining sunscreens with active agents (such as niacinamide, hydroxyphenoxypropionic acid, dipotassium glycyrrhizinate, glycolic acid, and 4-n-butylresorcinol) yielded positive results [55]. However, the studies conducted included small samples (*n* = 12), requiring randomised, double-blind clinical trials with representative samples to corroborate these results. The efficacy of sunscreen used alone is supported by several studies, although its protective effect may be insufficient to address all relevant wavelengths and mechanisms involved in pigmentation disorders [55]. In fact, a randomized double-blind study demonstrated that sunscreens effective against visible light and UV radiation promote the depigmenting effect of hydroquinone, supporting its combined use, according to Sarkar, R., et al. [66]. Similarly, Ko, D., et al. [64] highlighted the synergy of combining topical therapies with targeted photoprotection, citing a study where the adjunct use of 4% hydroquinone with a sunscreen providing both UV and visible light protection led to a 78% decrease in MASI scores, compared to a decrease of 62% in the group using 4% hydroquinone with a sunscreen protecting against UV radiation alone. Furthermore, it is also important to highlight the importance of sun protection during pregnancy, a phase especially sensitive to melasma relapses. A study with 185 pregnant women in Morocco reported that the use of broad-spectrum sunscreen with SPF greater than 50 reduced the rate of new melasma cases from 53% (obtained in a previous study) to just 2.7%, corresponding to 5 new cases [63,64].

Regarding post-inflammatory hyperpigmentation, studies suggest that the use of broad-spectrum sunscreens can help prevent exacerbation and contribute to better therapeutic outcomes, especially in the first week after dermatological procedures, such as laser therapy [57]. This recommendation is corroborated by Passeron, T., et al. [63]. This review, along with Ko, D., et al. [64], also highlights that preventing PIH during early epidermal healing requires strict light exclusion; specifically, the use of opaque dressings to completely shield the skin from all solar radiation is recommended during the first 15 days post-injury, with rigorous topical sun protection being essential if solar radiation exposure cannot be entirely avoided [63,64].

On the other hand, the combination of sun protection with anti-inflammatory and sebum-reducing agents did not obtain significantly different results from those achieved with the isolated use of sunscreen [60]. McDonald, K. A., et al. [19] reinforce the use of the compound terephthalylidene dicamphor sulfonic acid, a filter against UVA radiation, and drometrizole trisiloxane, a UVA and UVB filter, was associated with greater protection against post-inflammatory hyperpigmentation. Furthermore, McDonald, K. A., et al. [19] emphasise the importance of recommending broad-spectrum sunscreen with UV and visible-light protection for all individuals, of any phototype, with hyperpigmentation. However, it does not mention the greater susceptibility of Fitzpatrick phototypes IV to VI to these wavelengths, and the consequent need for greater photoprotection [19].

On the other hand, vitiligo has a complex, dual relationship with solar radiation. While the worsening phases may be associated with excessive or uncontrolled exposure to solar radiation, its repigmentation largely depends on phototherapy or heliotherapy. Natural sunlight exposure should not be considered equivalent to phototherapy or as requiring exposure until erythema develops. For this reason, sun protection becomes essential to ensure the balance between inducing repigmentation and preventing cumulative damage consequent to sun exposure [31,32,34]. It is, therefore, advisable for vitiligo patients to use broad-spectrum sunscreen with SPF 50+, as recommended by Passeron, T., et al. [63].

However, it is important to highlight that currently available studies present some obstacles to their practical applicability. One of the main challenges identified is the discrepancy between the ideal sunscreen application conditions used in clinical trials (2.0 mg/cm^2^) and the actual sunscreen use by consumers (0.39–1.0 mg/cm^2^). This difference considerably reduces the level of photoprotection achieved and, consequently, the sunscreen’s efficacy in everyday situations. This factor should be considered when interpreting the clinical results of these studies and in formulating practical recommendations for patients with these conditions. Future studies should evaluate the efficacy of sunscreens based on the amounts usually applied by the population to formulate more reliable practical recommendations. Although innovative filters, such as MCE, show promising results, the literature still lacks robust long-term studies that evaluate their efficacy in different phototypes, environmental contexts, and usage patterns. Furthermore, there is also a need to directly compare the performance of these new formulations with sunscreens currently on the market under real-world usage conditions. The association of anti-inflammatory and antioxidant active ingredients with filters protecting against UVA1 and visible light is also highlighted as having additional benefits in preventing relapses and controlling lesion progression, as briefly mentioned by Abdel Azim, S., et al. [65]. On the one hand, anti-inflammatory compounds can prevent UV light-mediated inflammation and ROS by decreasing neutrophilic inflammation and inhibiting proteases induced by UV radiation and ROS. Anti-inflammatory compounds, whether steroidal or non-steroidal, inhibit the production of inflammatory agents such as prostaglandins, leukotrienes, and inflammatory cytokines. On the other hand, topical antioxidants can prevent the production of ROS and subsequent inflammatory reactions, reduce UV radiation-induced skin damage and prevent the photoaging process itself [14].

From a clinical perspective, this review reinforces the importance of personalising photoprotection strategies for addressing individuals with pigmentation disorders. It is necessary to adapt the choice of sunscreen not only to the individual’s condition but also to the SPF, spectral coverage, cosmetic acceptability, and patient adherence profile. As McDonald, K. A., et al. [19] concluded, the most appropriate sunscreen formulation depends on various factors. Therefore, it is essential to personalise sun protection choices to the individual to improve adherence and subsequent clinical outcomes. Healthcare professionals play an important role in this context, as they can facilitate an informed choice by advising their users on the benefits and potential adverse effects of various sunscreen formulations and on their appropriate use. Consequently, promoting photoprotection literacy—including frequent reapplication, the use of an adequate dose, and the choice of products with effective coverage for UVA1 and visible light—can have an impact on the quality of life of patients with skin pigmentation disorders comparable to the development of new sunscreen filters.

However, the interpretation of the results must consider the heterogeneity in sunscreen formulations and the populations studied. Attributing the observed differences in the photoprotective efficacy of a given commercial product is also complicated by the presence of complex compositions involving multiple active ingredients with their own characteristics, making it difficult to establish a causal relationship between each ingredient and the observed effect. In addition, potential industry sponsorship and conflicts of interest should be considered when interpreting results, particularly in studies evaluating specific commercial products. Although industry funding does not necessarily compromise the validity of the results, the limited number of independent comparative studies represents a significant limitation of the current evidence base that should not be overlooked.

## 10. Conclusions

The present review reinforces the importance of sun protection as a fundamental measure in the prevention and adjuvant treatment of cutaneous pigmentation disorders. More specifically, the role of the daily use of broad-spectrum sunscreen is highlighted in reducing hyperpigmentation and improving the clinical appearance of pre-existing lesions. Protection against UVA1 radiation and visible light, particularly blue light (380–455 nm), is especially relevant in these disorders, as these wavelengths have been associated with the induction and exacerbation of hyperpigmentation. Special care may be warranted for individuals with higher Fitzpatrick skin phototypes (IV–VI), as they present a greater predisposition to develop hyperpigmentation induced by blue light and long UVA. Pigmented sunscreens containing iron oxide or coloured pigments may provide enhanced protection against visible light compared with some untitled formulations, while also offering the cosmetic benefit of camouflaging existing hyperpigmentation. However, aesthetic barriers may limit some consumers’ acceptance and consistent use. Therefore, despite significant advances in the last few decades, important limitations persist—namely, the discrepancy between the doses of sunscreen applied in clinical trials and those used by consumers, as well as the scarcity of long-term studies and those directly comparing the efficacy of new formulations with sunscreens currently available on the market. Attributing a specific photoprotective effect to a particular active ingredient can be difficult due to the different commercially available formulations that hinder this process. Potential industry sponsorship and the resulting conflicts of interest can also introduce bias into this interpretation and should not be overlooked. Furthermore, the discrepancy between the doses of sunscreen applied in clinical trials and those used by consumers in everyday life remains a significant limitation for translating the results into clinical practice. As a result, photoprotection should be considered a fundamental measure in the management of pigmentation disorders. In the future, the combination of broad-spectrum filters, visible-light protection, and antioxidant and/or anti-inflammatory agents should be considered, simultaneously reinforcing education in photoprotection and clinical research in real-world consumer sunscreen use contexts.

## Figures and Tables

**Table 1:** Summary of the epidemiological studies relating sun exposure and sunscreens.

Country	Study Type	Population	Sunscreen Type	Main Results	Reference
Romania	Monocentric, randomized, double-blind comparative prospective study	Included 20 volunteers, 10 women and 10 men, with a mean age of 27.5 years; 10 with Fitzpatrick phototype III and 10 type IV.	Two sunscreen formulas were tested. The reference was composed of 5% octocrylene; 1.5% avobenzone; 1% bis-ethylhexyloxyphenol methoxyphenyl triazine; and 0.3% terephthalylidene dicamphor sulfonic acid. The tested formula contained 5% octocrylene; 0.8% avobenzone; 1.1% bis-ethylhexyloxyphenol methoxyphenyl triazine; 1.5% terephthalylidene dicamphor sulfonic acid; and 1.5% MCE.	The addition of MCE in sunscreens extended absorption up to 400 nm, reduced UVA1-induced skin damage, and decreased sun-induced pigmentation.	[48]
France	Monocentric randomised double-blind prospective study	Included 20 healthy participants (10 women and 10 men, mean age 37 ± 8 years) from France. Of these, 15 individuals had Fitzpatrick phototype IV, and 5 type V.	Three products were tested: Product A: Anti-Dark Spots, SPF 50; composed of octisalate, ethylhexyl triazone, bis-ethylhexyloxyphenol methoxyphenyl triazine, and avobenzone. Product B: BB solaire, SPF 50; composed of titanium dioxide, nano-particulated titanium dioxide, homosalate, octisalate, ethylhexyl triazone, bis-ethylhexyloxyphenol methoxyphenyl triazine, drometrizole trisiloxane, avobenzone, octocrylene, and terephthalylidene dicamphor sulfonic acid. Product D: Dermablend Fluid; composed of titanium dioxide associated with iron oxide.	Repeated exposure to visible light induces skin pigmentation, and the tested products offer different levels of protection against this effect. Product D showed the highest efficacy, significantly reducing pigmentation compared to the other products and the untreated area.	[39]
Brazil	Prospective study	Included 40 women, aged 18 to 39, with Fitzpatrick phototypes II-III and brown hyperpigmentation macules on the face, 2 to 6 mm in size.	Two sunscreens were tested, one with only sunscreen filters and another with added pigments, both with SPF 30. Sunscreen filters: butyloctyl salicylate, titanium dioxide, alumina, hydrogenated dimethicone, polyhydroxystearic acid, isopropyl titanium triisostearate, and triethoxycaprylylsilane at 9%, and zinc oxide, butyloctyl salicylate, C12-15 alkyl benzoate, and triethoxycaprylylsilane at 8.5%. Pigments: yellow, red, black, and white; including synthetic wax, CI77492, CI77491, CI77499, isopropyl titanium triisostearate, and titanium dioxide.	Protection against visible light reduces skin hyperchromias by decreasing melanin synthesis (due to pigments associated with sunscreen) and facilitating cell renewal.	[38]
United Kingdom	Monocentric prospective study	Included 10 participants with Fitzpatrick phototypes IV to VI from the United Kingdom.	No photoprotection products were tested.	There is a synergistic relationship between visible light and UVA1. The combination of visible light and UVA1 caused pigmentation of greater intensity and clinical erythema, which was not observed with visible light alone. Reciprocity was only observed in areas exposed to visible light. The variation in the radiation spectrum had a greater impact on pigmentation intensity than irradiance. It is necessary to develop photoprotection means against visible light.	[35]
Germany	Prospective study	Included volunteers with Fitzpatrick phototypes II, IV, and V.	No photoprotection products were tested.	All spectra of solar radiation (UV, visible, and infrared) induce the formation of free radicals in Fitzpatrick phototypes II, IV, and V. After 4 min of simulated sun exposure, in the UV and IV spectrum, radical formation in skin types IV and V was 60% of that observed in type II, indicating that people with darker skin also need adequate sun protection.	[51]
Switzerland	Randomised, double-blind, placebo-controlled prospective study	Included 33 women, with Fitzpatrick phototypes III and IV.	No photoprotection products were tested. The tested products consisted of a base formula (composed of water, sodium gluconate, propanediol, xanthan gum, cetearyl and sorbitan olivate, cetearyl alcohol, phenoxyethanol, ethylhexylglycerin, caprylic/capric triglyceride, octyldodecanol, dimethicone, hydroxyethyl acrylate/sodium acryloyldimethyl taurate copolymer), to which 3% Scenedesmus rubescens microalgae extract was added in one product and 3% niacinamide in another.	Blue light induces skin changes, such as alterations in skin chromophores, erythema, hyperpigmentation, and other signs of photoaging. These effects were mitigated by both the formula containing 3% Scenedesmus rubescens microalgae extract and the formula with 3% niacinamide.	[43]
Brazil and China	Randomized comparative prospective study	Included 113 women from Brazil and China. In Brazil, 53 women, aged between 35 and 65, with Fitzpatrick phototypes I to III were included. In China, 60 women, aged between 25 and 65, with Fitzpatrick phototypes III and IV were included.	An SPF 50 sunscreen and the same sunscreen enriched with 3% MCE were tested.	The same SPF 50 sunscreen significantly reduces pigmentation and signs of photoaging when enriched with MCE.	[49]
USA	Monocentric, randomised, single-blind prospective study	Included 10 individuals, with a mean age of 35 ± 6 years, with Fitzpatrick phototype IV.	Three sunscreens were tested. Product A consisted of a mineral sunscreen with SPF 50+ containing 6.5% zinc oxide and 4.5% titanium dioxide. Product B consisted of a formulation with 4.85% iron oxide and 27% titanium dioxide. Product C consisted of a formulation with 27.25% iron oxide.	Formulations with iron oxide significantly protect against visible light-induced pigmentation, possessing dual benefits by masking existing pigmentation and preventing the development of new sun-induced spots, in addition to UV protection.	[40]
Peru	Prospective study	Included 17 volunteers from Peru of all Fitzpatrick phototypes.	10 sunscreens were tested, with SPF ranging from 30 to 110, with broad-spectrum UV and UVA filters.	Despite claims of high protection (SPF 30–110) and broad-spectrum, 97% of sunscreen-protected areas still suffered sun damage (erythema and/or pigmentation) after less than 2 h of natural sun exposure. Furthermore, sunscreens with only FDA-approved filters performed worse.	[52]
United Kingdom	Monocentric prospective study	Included 9 volunteers (6 men, 3 women) with a mean age of 25.6 ± 3.2 years, with Fitzpatrick phototype I and II.	Two sunscreens, SPF 15, identical except for the addition of bi-(ditelamino-hydroxybenzoylbenzoyl)piperazine (BDBP) were tested.	The sunscreen formulation with the BDBP filter provides significantly greater photoprotection.	[36]
France	Randomised prospective study	Included a total of 62 healthy individuals with Fitzpatrick phototypes III and IV. These were divided into 3 groups. 1st group: 20 individuals, European, 15 women and 5 men, mean age 25.4 years; 8 with Fitzpatrick phototype III and 12 type IV. 2nd group: 24 individuals, European, 10 women and 14 men, mean age 24.8 years; 10 with Fitzpatrick phototype III and 14 type IV. 3rd group: 18 individuals, Indian, 18 women, mean age 24.4 years; all with Fitzpatrick phototype IV.	An SPF 30 sunscreen and the same sunscreen enriched with 2% methoxypropylamino cyclohexenylidene ethoxyethylcyanoacetate (MCE) were tested.	In the evaluated formulation, sunscreen with 2% MCE showed better prevention of hyperpigmentation compared to conventional sunscreen, extending protection against UVA1 without reported side effects.	[50]
Mauritius	Monocentric, open, randomised prospective study	Included 20 women, aged 18 to 50, with Fitzpatrick phototypes III to V.	9 sunscreens, SPF 50+, containing the broad-spectrum UV and blue light filter phenylene bis-diphenyltriazine (PBDT or TriAsorB™) and three other organic UV filters (bis-ethylhexyloxyphenol methoxyphenyl triazine, diethylamino hydroxybenzoyl hexyl benzoate, and ethylhexyl triazone), which differed from each other in fragrance and consistency. Two of these products were selected for in vivo evaluation: one untinted and one tinted.	All tested products were highly photostable and photoprotective against blue light. In vivo, they also prevented immediate skin pigmentation.	[44]
USA	Monocentric, open, randomised prospective study	Included 12 volunteers (10 women and 2 men), with a mean age of 32.5 years. Of these, 8 had Fitzpatrick phototype III and 4 type IV.	Four sunscreens were tested. Product A: SPF 60, broad-spectrum, 3% avobenzone, 10% homosalate, 5% octisalate, 7% octocrylene. Antioxidants: tocopherol and Cassia alata leaf extract. Contains no iron oxide. Product B: SPF 50, broad-spectrum, 15% homosalate, 5% octisalate, 7% octocrylene, 12% zinc oxide. Antioxidants: tocopherol and Cassia alata leaf extract. 4% iron oxide. Product C: SPF 50, broad-spectrum, 15% homosalate, 5% octisalate, 7% octocrylene, 12% zinc oxide. Antioxidants: tocopherol and Cassia alata leaf extract. Contains no iron oxide. Product D: SPF 50, broad-spectrum, 11% micronised titanium dioxide. Antioxidants: tocopherol and Cassia alata leaf extract. 1% iron oxide.	Product D showed better and more consistent protection across all evaluated parameters (erythema and pigmentation), whereas Product B also showed some efficacy. Among the products evaluated, tinted products provided greater protection against visible light and UVA1 than untinted ones. The product with 1% iron oxides showed greater protection than the product with 4% iron oxides in this study; however, these findings should not be interpreted as evidence that a lower concentration of iron oxides is generally more effective, as differences in the overall formulation may have contributed to the observed effects. Further comparative studies are needed.	[41]
Brazil	Randomised prospective study	Included 52 Brazilian women, with a mean age of 52.2 ± 7.8 years. 2 with Fitzpatrick phototype I, 22 type II, and 28 type III.	An SPF 50 sunscreen and the same sunscreen enriched with 1% MCE were tested.	In the evaluated formulation, sunscreen with 1% MCE showed better protection against hyperpigmentation, erythema, and signs of photoaging compared to the same sunscreen without MCE, suggesting a potential benefit of extended protection against UVA1 radiation.	[37]

**Table 2:** Summary of the in vitro studies on sun exposure, photoprotective substances, and mechanisms of skin damage.

Photoprotective Agent/Tested Substance	Experimental Model/Cell Type	Main Results	References
33 sunscreens available on the Brazilian market, with SPF between 30 and 99, were tested.	Each product was applied to 4 polymethyl methacrylate plates, in the amount of 1.3 mg/cm^2^.	Sunscreens with pigments showed a higher sun protection factor in the visible light range and a higher pigmentation protection factor compared to products without pigment. There is a strong correlation between the visible light protection factor and the pigmentation protection factor.	[42]
No photoprotection products were tested.	Immortalised keratinocytes from the HaCaT cell line were used.	The long UVA radiation spectrum has been associated with several mechanisms of photodamage, including cell viability, DNA damage (delay of cyclobutane pyrimidine dimers), increased gene expression of genes associated with inflammation, oxidative stress, and photoaging, and induction of oxidative species in HaCaT keratinocytes and in skin in vivo.	[47]
The aqueous extract of Polypodium leucotomos (Fernblock^®^) was tested.	Human fibroblasts and murine melanocytes were used.	Pre-treatment with Fernblock^®^ prevented cell death, mitochondrial morphology alteration, and p38 phosphorylation in human dermal fibroblasts exposed to blue light. Furthermore, it significantly reduced opsin-3 activation in melanocytes and melanin photo-oxidation, preventing its photodegradation.	[45]

**Table 3:** Summary of the epidemiological studies relating melasma and sunscreens.

Country	Study Type	Population	Sunscreen Type	Main Results	Reference
France	Randomized comparative prospective study	Included 39 participants, who were divided into 2 groups. Group A consisted of 18 women and 1 man, with a mean age of 43 ± 9.0 years; of these, 5 had Fitzpatrick phototype III, 9 type IV, and 5 type V. Group B consisted of 19 women and 1 man, with a mean age of 41.4 ± 7.3 years; of these, 13 had Fitzpatrick phototype III, 6 type IV, and 1 type V.	Two sunscreen formulas were tested. Both contained the same sunscreen filters: octocrylene, methylene bis-benzotriazolyl tetramethylbutylphenol, butyl methoxydibenzoylmethane, bis-ethylhexyloxyphenol methoxyphenyl triazine, and titanium dioxide. Formula A was tinted and also contained iron oxide.	Formula A (tinted and with iron oxide) provided greater protection against melasma relapses than Formula B (colorless). Twelve participants used Formula B and tinted makeup with no difference in the result obtained compared to the remaining participants using Formula B, suggesting that protection against short-wavelength visible light is more relevant than simple tinting. Spectrophotometric analysis indicated that the protection offered by the tinted sunscreen was partial, highlighting the need for more effective filters.	[46]
India	Randomized controlled double-blind prospective study	Included 4 men and 42 women, aged 25 to 50, with moderate to severe hypermelanosis, consistent with a diagnosis of melasma.	An SPF 30 sunscreen with a physical sunscreen filter, composed of 2% octocrylene, 2% octyl methoxycinnamate, 7.5% oxybenzone, and 2% zinc oxide. It was compared with a combination of the same sunscreen with phenylethyl resorcinol, nonapeptide-1, aminoethylphosphinic acid, and antioxidants.	The combination of compounds with sunscreen improved the melanin index, but without statistical significance when compared to the isolated use of sunscreen. The melasma area and severity index decreased with the combination and increased with the isolated use of sunscreen. The combined formula was associated with better maintenance of melasma remission.	[56]
USA	Randomised double-blind prospective study	Included 30 women diagnosed with melasma for more than 2 years, over 18 years old, who were not using sunscreen.	A sunscreen with SPF 50+ was tested, containing benzophenone-3, octinoxate, octocrylene, titanium dioxide, zinc oxide, and iron oxide. From this, 3 samples were composed: sunscreen plus 4% niacinamide, sunscreen plus 0.05% retinoic acid, and sunscreen plus placebo.	Sun-induced DNA hypermethylation was identified in melasma lesions. Treatment with isolated sunscreen and combined with niacinamide or retinoic acid reduced methylation, correlating with clinical improvement.	[53]
India	Double-blind prospective study	Included 100 participants (80 women and 20 men), with a mean age of 33.3 ± 6.7 years, diagnosed with melasma, untreated in the last 3 months, with Fitzpatrick phototypes III and IV.	Garnier White Complete sunscreen with SPF 19 and PA+++ was tested; it was composed of octocrylene, butyl methoxydibenzoylmethane, drometrizole trisiloxane, phenylbenzimidazole sulfonic acid, and sodium acryloyldimethyl taurate.	There was a statistically significant improvement in both the participants’ quality of life and MASI after 12 weeks of sunscreen treatment.	[54]
Italy	Monocentric prospective study	Included 12 Caucasian women with melasma, aged 30 to 60, with Fitzpatrick phototypes II to IV.	An SPF 50+ sunscreen was tested, associated with niacinamide, hydroxyphenoxypropionic acid, dipotassium glycyrrhizinate, glycolic acid, and 4-n-butylresorcinol.	A topical skin lightening serum, composed of niacinamide, hydroxyphenoxypropionic acid, dipotassium glycyrrhizinate, glycolic acid, and 4-n-butylresorcinol, and SPF 50+ sunscreen, demonstrated rapid clinical efficacy in improving melasma hyperpigmentation, with good tolerance.	[55]

**Table 4:** Summary of the clinical and histopathological studies relating on post-inflammatory hyperpigmentation (PIH) and the impact of photoprotection.

Country	Study Type	Population	Sunscreen Type	Main Results	Reference
India	Monocentric, single-blind, uncontrolled prospective study	Included 58 men and 172 Indian women, with facial pigmentation changes (actinic lentigines and post-inflammatory hyperpigmentation), with Fitzpatrick phototypes IV and V.	Two products were tested. Product A: Vichy Capital Soleil Dry Touch, SPF 50 and PA+++; composed of homosalate, ethylhexyl salicylate, ethylhexyl triazone, bis-ethylhexyloxyphenol methoxyphenyl triazine, drometrizole trisiloxane, avobenzone, octocrylene, and titanium dioxide. Product B: Garnier White Complete, SPF 19 and PA+++; composed of titanium dioxide, octocrylene, and avobenzone.	Regular use of sunscreen with high to moderate SPF and high PA+++ protects against pigmentary irregularities and improves skin luminosity in Indians with phototypes IV and V, reducing signs of photoaging.	[59]
South Korea	Prospective study	Included 20 men with Fitzpatrick phototype III or IV, with a mean age of 24.5 years.	No photoprotection products were tested.	Melanocyte activation after laser treatment causes increased skin pigmentation. To avoid this pigmentary change, intervention should occur at least 1 week after treatment.	[57]
South Korea	Retrospective study	Included 21 participants (10 men and 11 women) aged 1 to 60 years, 8 with Fitzpatrick phototype III and 13 type IV.	No photoprotection products were tested.	Post-inflammatory hyperpigmentation can be grouped into 2 histopathological groups: dermal and epidermal pigmentation. The group with dermal pigmentation has a lower level of epidermal pigmentation. This histopathological information can improve the treatment of post-inflammatory hyperpigmentation.	[58]
Thailand	Randomised double-blind prospective study	Included 59 individuals (42 women and 17 men), with a mean age of 28.56 ± 6.17 years, who were treated with 1 session of picosecond laser for acne vulgaris and moderate acne scars on the face. Of these, 33 had Fitzpatrick phototype III and 26 type IV.	Two sunscreens were tested. Sunscreen A: Eucerin broad-spectrum sunscreen, containing licochalcone A, L-carnitine, octocrylene, butyl methoxydibenzoylmethane (avobenzone/BMBM), and homosalate. Sunscreen B: sunscreen with the same filters, but without anti-inflammatory and sebum-reducing agents.	Sunscreen, in association with anti-inflammatory and sebum-reducing agents, showed a greater reduction in hyperpigmentation compared to sunscreen without these agents, but this difference was not statistically significant. This combination was more effective in reducing inflammatory acne lesions and porphyrin levels, with statistical significance.	[60]

## Data Availability

No new data were created or analyzed in this study. Data sharing is not applicable to this article.

## Abbreviations

The following abbreviations are used in this manuscript:

DNA	Deoxyribonucleic acid
DNMT	DNA methyltransferase
USA	United States of America
FDA	Food and Drug Administration
SPF	Sun protection factor
IR	Infrared
MASI	Melasma Area and Severity Index
MCE	Methoxypropylamino Cyclohexenylidene Ethoxyethylcyanoacetate
MMPs	Matrix metalloproteinases
PA	UVA protection grade scale
ROS	Reactive oxygen species
UV	Ultraviolet
